# Cases of Inhalation of Ether in Labor

**Published:** 1847-08

**Authors:** 


### ECLECTIC DEPARTMENT,

### AND SPIRIT OF THE MEDICAL PERIODICAL PRESS.

---

*Cases of Inhalation of Ether in Labor.* By WALTER CHANNING, M. D., Professor of Midwifery, Massachusetts Medical College.

Mrs. R., aged 18; first child. Taken with uterine pain June 10, at 5, A. M. Saw her between 3 and 4, P. M. Labor well marked—pains severe—complaint of suffering emphatic. Examination showed natural presentation, and good progress. Pains increased gradually in strength, and the head reached the outlet favorably. It here made slight, if any progress. At length, notwithstanding very severe pains, it hardly advanced at all. Between 8 and 9 it reached the external organs, and the perineum was pressed somewhat forward. Here again it rested. The perineum was very slightly dilatable. Pains now diminished in force, and the intervals became longer. Still distress was great. I sent for, and exhibited ether. The pulse, respiration, and temperature, were natural. At first Mrs. R., refused the sponge, and this with much determination. At length she consented to breathe it, and was in about a minute fairly under the influence of the vapor. The sponge was removed, and placed at a distance from her in the bed. Pains continued regular, and soon became stronger, and intervals shorter. The perineum became dilatable, and the head advanced. Some return of consciousness took place, it would seem of pain, and of the relief she had experienced from the inhalation, for without being seen she got possession of the sponge, and breathed at it with the greatest avidity, so that when discovered it was with much difficulty forced away. The child was born in four or five pains after etherization. The placenta was soon thrown off—the womb contracted well, and a swathe was carefully applied.

The return to consciousness was slow. There was exhibited more excitement than I have before met with. There was a full expression of previous most perfect freedom from suffering. A state of entire pleasure was expressed. She sung, talked, raised her arms high in the air. She did not recollect me, or anybody about her. Her child's cries, which were very loud, attracted strongly her notice. She passed her had over her abdomen firmly, as if to learn what had happened, and her countenance expressed much surprise. Pulse continued natural; complexion good; temperature, as during labor. Some hemorrhage, but not enough to do harm. She said she was very hungry, and thirsty, and took with much relish, gruel and water. After-pains occurred in about half an hour after labor was over, with much severity, and for which I prescribed camphor and opium in pills. I left her otherwise comfortable. Slight hemorrhage.

June 11th. 9, A. M.—Pulse, &c., good, no tenderness of abdomen. After-pains increased, and troubled her all night. Exist now. Great increase of pain by motion, so that I removed urine by catheter, as motion for this function she feared would give great distress. Felt better after operation. Hemorrhage considerable in night, but not sufficient to do harm. 6, P. M. More comfortable; less pain. Prescribed Dover's powder for pill. Ol. ric. in the morning.

12th. 6, P. M.—Detained by obstetric case all day, and could not see Mrs. R. before. I found her very comfortable. Oil has operated well. Night had been perfectly good. Looks remarkably well. Pulse good. Abdomen soft, free from soreness or pain.

14th.—Perfectly well. Some milk in breasts. I now inquired particularly as to consciousness during labor, and immediately after. Remembers nothing. Is surprised at questions, so wholly unconscious as they show her to herself, to have been.

June 12th.—Mrs. A W., 35; first labor. For three weeks last past, she has suffered much from pain referred to womb. At times so severe that she thought of sending for nurse, and physician. Last night, at 11, in absence of pain, membranes broke, and a large quantity of water was discharged. I was called to see her between 1 and 2, as pains had begun. I found the os uteri dilated somewhat, dilatable, very thin at its edges, and continuing so some distance towards neck. Pains regular but slight. I went to bed, to be called when needed. At 6, I found pains had continued all night. Os uteri more dilated. Went home. Called again between 8 and 9. I now found much change had occurred. Os uteri had nearly disappeared, and yielding to very small pressure Head presenting well, and quite low in pelvis. From the severity of the pains, and the generally favorable state of things, I resolved to use ether. This was done about 9, A. M. Its first effect was excitement. There were startings, exclamations, the arms were projected. "I am dying, I am dying," said Mrs. W. I had my finger on the wrist, and carefully examined the pulse. It was about 90 before etherization. It rose to 98; and this was its number, with temporary changes, during the whole of the labor after ether. Excitement soon passed by, and a pleasant calm succeeded. The expressions were now of pleasure only. "How beautiful! how beautiful!" was the language of the labor. The state of etherization was moderately sustained during the whole day. Mrs. W. had some latent feeling about the remedy which much influenced the case. She would vehemently demand the sponge, and that it should be thoroughly wet with ether. She would put it aside, as as soon as she began to feel its effects. At times, however, she would experience its full effects. She was thus by no means wholly unconscious. I mean in that degree of it as to be unaware of people and things around her. Sometimes she would say, "I know you, Dr. C.," "I know you, Mrs. F.," &c., &c.; as if to let us understand that though she was unconscious of pain, she knew all other things. She would say, when demanding the sponge "don't be afraid of hurting me. I know just how much I want, and will tell you when to take it away." And this was done after a manner which I have seen in no other case. The labor was delayed by the state of the perineum. It was very wide, leaving the os externum very small. Through this protruded a round mass of scalp, and a conical-shaped bony mass of skull. The occiput had fairly cleared the arch of the pubis, and still the delivery did not take place. After an ointment of ext. of belladonna and simple cerate was liberally used, inside the vagina, and over the perineum, dilatation took place readily, and the child was born. There was perfect abolition of pain in this closing period of labor, and when suffering is, I may say, always so great. The womb contracted well. The placenta was easily detached by natural effort, and, with some coagula, was expelled. A swathe was applied. Child, a female, weighing seven and a half pounds. It did not brethe immediately after head was born, but soon breathed after cold water was dashed on its face and breast, and did perfectly well. During the labor, ʒ j. ergot was infused in about ℥ vi. boiling water, and the tea without the powder taken. Some increase of pain undoubtedly followed its use. The bladder was emptied with the catheter once during the day.

Labor began in this case at 12 the preceding night, and was ended at 6, P. M., the following day. The ether was first inhaled between 8 and 9, A. M., and its influence was sustained, as above described, till nearly 6, P. M. Mrs. W. described her state, when consciousness returned after labor, as one of perfect ease and enjoyment. She had not a pain. She had little memory of pain. The ether had made tolerable what she though she could hardly have lived through without. She had been in labor, she said, three weeks; her nights disturbed and her days most uncomfortable. She expressed her gratitude for this means of her comfort after a manner which I have not heard paralleled. I left her with a calm pulse, manner perfectly natural, skin temperate, head free from pain, abdomen easy, eyes closed, and sleep approaching.

13th. 10, A. M.—Night good. Slight pain in abdomen in night occurred three times. Five grains of Dover's powder were given, and perfect quiet followed. Pulse 84; abdomen soft; lochia natural; no urine, has attempted to pass it, but failed. Catheter, about twenty ounces taken away. In all respects doing well. 10, P. M. Day very comfortable. Has slight uneasiness in the region of the heart, to which she has long been subject. Has failed to pass urine, catheter was used. Ol. ric., suc. limon, *aa* ʒij., M. in the morning.

14th. 9, A, M.—Excellent night. Urine natural. Two free dejections from oil. Pulse, &c., natural. Milk, without any precursory disturbance or excitement. Is nursing her child.

15th.—Quite as well, except some trouble about lactation.

June 13th.—Mrs. G. W. A., 36, fourth child. Labor began Friday, 11th. Physician called Saturday, 12th, at 8, A. M. Labor pains distinct, strong; head at upper part of pelvis, or rather a cushiony tumor of which the diagnosis was not easy. This state of things continued. He passed Saturday night with his patient because of the severity of the labor. Sunday, 13th, things much the same. Membranes broke P. M., and a large quantity of water came away. Still the head remained much where it was. The os uteri dilatable, soft, spongy, as if infiltrated. He passed the night again with his patient. Monday morning I saw her at 8, A. M., about sixty hours from beginning of labor, and forty-eight of continuous suffering.

The presentation was just what and where it was when first discovered. A firm, somewhat elastic tumor filled the pelvis. I did not, at my first examination, feel any portion of the cranum. The tumor felt very much like a blood tumor of great size. It remained tense in *the intervals* of pains, and did not seem more tense during pains. I advised the use of ether at once, to lessen the severe suffering of the patient, and having directed how it was to be used, left, to see a patient whose situation made an early visit very important. I returned between 8 and 9. Partial relief had been gained by the ether; suffering was less. I examined again with much care, and could make out towards the right sacro-iliac synchondrosis the well-defined *edge of a bone*. I pressed the presenting tumor here very firmly, and could find no bone opposite to or in the neighborhood of that against which my finger had rested. I now felt satisfied that the case was one of hydrocephalus, and that it was water which I felt behind the scalp, and which formed the tumor. The ether was used. Perfect unconsciousness did not take place more than once, but the diminution of suffering was most striking, and the ether more and more emphatically demanded. The perforator was now carried through the distended scalp and a gush of water at once followed. Nearly a quart was received into a vessel, while a very large quantity escaped into the bed and guard. Extraction was now made, and after a few ineffectual efforts, the head advanced. The difficulty was in the loose condition of the bones, and the thinness of the scalp. The hook, from these circumstances, frequently broke itself away. The ether was still used. Entire abolition of consciousness was produced. The pulse continued steadily at about 100, the number when first examined. If anything it was quicker at my first visit than during the use of the ether; and while extraction was proceeding. The child was born slowly, and easily after the head had fairly entered the pelvis. The placenta followed readily. A swathe was applied, and the patient made easy and comfortable in her bed. Child's head consisted of loose cranial bones, thin scalp, and a large cavity. Spina bifida at lower part of spine. It seemed impossible for Mrs. A. to express the gratitude she felt for the pleasure and the ease afforded to her by the ether. It was astonishing to her, that she who had always suffered so much in labor, and for so many nights and days in this last one, and who after former labors had been in such distress—it seemed most wonderful to her to feel now so easy and so happy. I left her in this state at about 11, A. M.

5, P. M.—Saw Mrs A. She has been perfectly comfortable all day, has slept much, passed water twice, no after pains; much meteorism, but no hardness, soreness or pain of abdomen. Respiration easy, countenance easy, and has lost that contraction which the long experience of acute pain gave it, and has acquired the appearance of healthy fulness. Complexion natural. Pulse 108, soft. Says she is perfectly free from uneasiness of any kind. Is provisionaly to take ℥ij. ol. ric. and suc. lim. each, in the morning, and five grains pulv. Dover, if need be, to-night.

15th. 10, A. M.—Pulse 108. Abdomen less full. Respiration easy. Temperature natural. Night good. Has taken oil. No dejection.

16th. 9, A. M.—Pulse 104. Two dejections. Urine natural Without any uneasines swhen at rest. Is annoyed by motion, the whole body being sore, from long-continued and violent labor.

*Remarks*.—Mrs. R.'s case presents the full effects of ether perhaps more strikingly than either of the others. That is, in her they were more perfectly produced. Still the time was short of their continuance, and not any cause for uneasiness marked any of its periods. The sponge which she reached and held with so much force had become almost or quite dry, as some time had passed from its first application. Her case is also striking as presenting perhaps the most perfect want of memory met with in any case I have witnessed.

Mrs. W.'s case. In this case ether was used nearly or quite nine hours. But except in its first inhalation, and the latest, nothing like its full effect was manifested. She managed the use of it herself. That is, she asked for it when she thought it was needed, namely, when a pain was coming on, and threw it by her as soon as she felt its influence approaching. Again and again she has assured me that her suffering after etherization was as nothing compared with her former state, that the *last pains were not felt at all*. The case differs from others in the whole agency of the patient during this long trial of ether. I have never known so much used, and certainly its effects could not have been more happy. The consciousness of the period when ether was used had been matter of distinct memory since. There were misgivings among her friends as to the expediency of its use, for they knew that she had formerly suffered severely from headache; but this was never stated to me. She had suffered also from pain in the heart; but of this also, I knew nothing till it occurred slightly the day after labor, when it was referred to by Mrs. W. It is suggested that it will always be well to learn if peculiarities exist in patients, or if morbid predispositions may be supposed to belong to them. In the above case, however, nothing occurrrd for a moment to disturb the feeling of the entire safety of the patient.

Mrs. A.'s case differs from all the others. Bnt its history leaves very little to be added. Relief of suffering was as marked in her case as in any I have met with. I have never seen insensibility more strikingly produced by ether. She was for a short time as in the deepest, most tranquil sleep. This was at the close of the labor. It saved her all pain in the time of ordinarily the greatest suffering. She is recovering, though less rapidly than the others, but still quite as fast as the early history of her case would have led any one to expect.—*Boston Medical and Surgical Journal.*

